# Electrolytic ablation enables cancer cell targeting through pH modulation

**DOI:** 10.1038/s42003-018-0047-1

**Published:** 2018-05-17

**Authors:** Nicholas R. Perkons, Elliot J. Stein, Chike Nwaezeapu, Joseph C. Wildenberg, Kamiel Saleh, Roni Itkin-Ofer, Daniel Ackerman, Michael C. Soulen, Stephen J. Hunt, Gregory J. Nadolski, Terence P. Gade

**Affiliations:** 1 Penn Image-Guided Interventions Laboratory, 421 Curie Boulevard, BRB II/III, Philadelphia, PA 19104 USA; 2 Perelman School of Medicine, 3400 Civic Center Boulevard, Bldg. 421, Philadelphia, PA 19104 USA; 3 Department of Bioengineering, 210S 33rd St., Suite 240 Skirkanich Hall, Philadelphia, PA 19104 USA; 4 Department of Radiology, Hospital of the University of Pennsylvania, 3400 Spruce Street, Philadelphia, PA 19104 USA; 5 Department of Cancer Biology, 421 Curie Boulevard, BRB II/III, Philadelphia, PA 19104 USA

## Abstract

Minimally invasive ablation strategies enable locoregional treatment of tumors. One such strategy, electrolytic ablation, functions through the local delivery of direct current without thermal effects, facilitating enhanced precision. However, the clinical application of electrolytic ablation is limited by an incompletely characterized mechanism of action. Here we show that acid and base production at the electrodes precipitates local pH changes causing the rapid cell death that underlies macroscopic tumor necrosis at pH > 10.6 or < 4.8. The extent of cell death can be modulated by altering the local buffering capacity and antioxidant availability. These data demonstrate that electrolytic ablation is distinguished from other ablation strategies via its ability to induce cellular necrosis by directly altering the tumor microenvironment. These findings may enable further development of electrolytic ablation as a curative therapy for primary, early stage tumors.

## Abstract (web summary)

Nicholas Perkons et al. investigate the mechanism by which electrolytic ablation induces cell death in the localized treatment of tumors. They find that electrolytic ablation alters the pH buffering capacity of the tumor microenvironment to induce cellular necrosis.

## Introduction

Tissue ablation—a technique used to destroy pathological tissues—is one of several locoregional treatments used in the management of a variety of cancers, most commonly hepatocellular carcinoma (HCC). Ablation is distinguished among locoregional therapies by its capability to effect a cure for solitary, primary lesions^1,2^. Ablation modalities can be classified by their primary mechanism of action including thermal-dependent and thermal-independent modalities. Thermal-dependent modalities include radiofrequency ablation, microwave ablation, laser interstitial therapy, high-intensity focused ultrasound, and cryoablation. The most commonly utilized thermal-independent modality for tissue ablation is irreversible electroporation (IRE)^1,3,4^. Choice of modality in the clinic is primarily determined by the site of a lesion and the desired mechanism of cellular injury^5^.

Among the thermal-dependent modalities, the majority (radiofrequency ablation, microwave ablation, laser interstitial therapy, and high-intensity focused ultrasound) deposit energy, which causes hyperthermia and subsequent cell death through direct and indirect injury. Direct injury describes the nearly immediate effect of locoregional heat application at or above 60 °C. Indirect injury describes the disruption of normal cellular processes, leading to delayed cell death^4^. At temperatures above 42 °C, cell injury occurs more frequently in tumor cells than healthy cells with higher temperatures significantly increasing this therapeutic ratio and decreasing the requisite ablation times^6^. Analogous to the temperature dependence of hyperthermic ablation modalities, cryoablation relies upon cooling tissue below −40 °C to induce cell death^3,4^. At temperatures below freezing, ice forms in either the intracellular or extracellular space, inducing osmotic gradients that damage the integrity of the cell membrane^3,7^.

While an understanding of their mechanisms has led to the clinical application of these technologies for locoregional cancer therapy, their efficacy has been mitigated by important intrinsic limitations of thermal ablation. The primary limitation of thermal ablation is poor precision in defining the zone of ablation. Vessels traversing an ablation zone serve as heat sinks or sources, which can distort the temperature gradients within the ablation zone and lead to undesirable treatment margins^3,4^. This imprecision, in combination with safety considerations stemming from off-target toxicity, emphasizes the importance of developing non-thermal ablation strategies to treat cancer.

In comparison to thermal-dependent modalities, IRE kills cancer cells by disrupting membrane integrity^8,9^. IRE applies microsecond pulses of high electric potential (up to 3000 V) between two or more electrodes^3^. While the tendency for heat to be generated scales with the amplitude of the voltage applied, IRE does not mechanistically depend on hyperthermia to cause cell death^8^. It is believed that this cell death instead proceeds from the induced transmembrane potential which irreversibly disrupts the integrity of the lipid bilayer; specifically, a potential of 1–2 V across a cell membrane is required for cell death to occur^10–13^. A distinct benefit of this technique is that the extracellular matrix remains mostly intact. The primary drawbacks of IRE are secondary side effects associated with the high magnitude of the applied voltages. The voltages of the delivered pulses have the potential to induce cardiac arrhythmias and muscle contractions, which necessitate the use of general anesthesia^3,14^. Furthermore, precise electrode alignment is required to ensure adequate charge deposition and to mitigate thermal injury to non-target tissues^3,14,15^.

Electrochemotherapy and gene electrotransfer are techniques that are related to IRE but are distinguished by their use of either fewer electrical pulses or lower voltage magnitudes, respectively. These modalities induce a temporary and sublethal permeabilization of cell membranes that facilitates delivery of cargo to cells. Electrochemotherapy is used in cancer therapy to enhance uptake of chemotherapeutic agents, such as bleomycin or cisplatin. Gene electrotransfer is analogous to electrochemotherapy, but instead facilitates the delivery of a gene or genes to cells to enable the downstream production of a therapeutic protein^16–18^. While these techniques demonstrate similar limitations to those seen with IRE, their utility issues from their selectivity and well-characterized mechanisms of action.

Electrolytic ablation, also known as electrochemical treatment, causes necrosis through the application of a direct current between multiple electrodes at relatively low electric potentials compared to IRE, often less than 50 V. In doing so, this technique offers unique advantages compared to other ablation methods^19^. Specifically, electrolytic ablation may permit the creation of precisely defined, shapeable ablation zones, that are uninfluenced by heat sink effects and can be monitored and adjusted in real-time using magnetic resonance imaging^20–23^. Electrolytic ablation has been utilized for the treatment of a variety of human malignancies; however, its development has been limited by uncertainty regarding the underlying mechanism of the induced cell death^19,24–26^. While it is well established that electrolytic ablation induces electrolysis, a variety of potential mechanisms have been identified that may underlie the observed cell death. These include deposition of electric charge, the creation of a cellular transmembrane potential, production of toxic substances, extraction of water through electroosmosis, and alteration of microenvironmental pH^27–38^. Mechanistic understanding of the cell death induced by electrolytic ablation is required to enable its further development for widespread clinical application. Furthermore, understanding cellular changes induced by electrolytic ablation will enhance parameter selection for related electrochemical therapies, including IRE, electrochemotherapy, and gene electrotransfer, which may have synergistic effects when combined with electrolytic ablation^39,40^.

In this study, we endeavored to define the mechanism by which electrolytic ablation causes cell death using an in vitro cell-encapsulation assay. After embedding living HCC cells in low melting temperature agarose, electrolytic ablation was performed, and patterns of cell death were measured. We demonstrate that following sustained exposure to a low amplitude direct current, cell death from electrolytic ablation proceeds primarily via a pH-dependent mechanism. Importantly, temperature change and transmembrane voltage gradient do not explain the observed cell death, confirming a non-thermal ablation mechanism that is distinct from IRE. These findings confirm earlier in vitro and in silico observations of pH changes following electrolysis during electrolytic ablation and gene electrotransfer^35,40,41^ and hold important implications for the development of electrolytic ablation as an alternative to existing ablation strategies.

## Results

### In vitro assay development

**Fig. 1:**
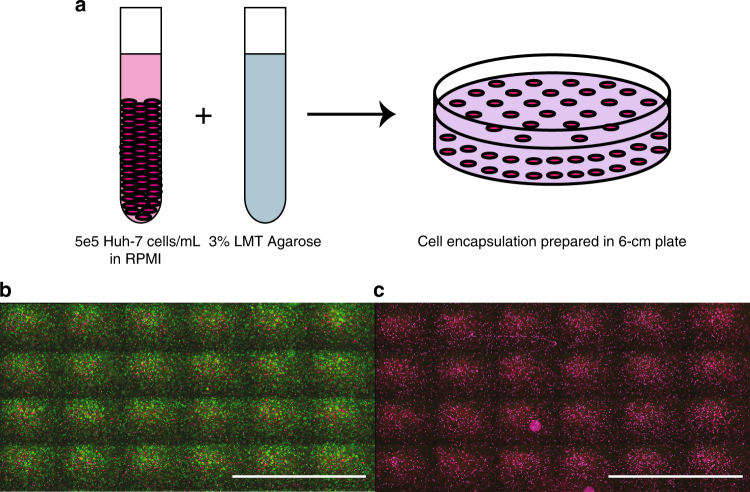
Model system: HCC cells embedded in low melting temperature agarose with viability assessment using a dual reporter assay. **a** The cell-encapsulation matrix was prepared as a 1:1 mixture of Huh-7 HCC cells and 3% low melting temperature agarose. **b** Staining with a pair of fluorescent viability reporters demonstrates that the encapsulation procedure does not impair cell viability (green fluorescence). Scale bar = 5 mm. **c** Boiling of the preparation resulted in complete cell death (red fluorescence (recolored to appear magenta) in the absence of green fluorescence). Scale bar = 5 mm

To evaluate the mechanism of cell death in electrolytic ablation, we developed an in vitro cell encapsulation assay in which HCC cells (Huh-7, unless otherwise specified) were embedded in low melting temperature agarose (Fig. 1a). We confirmed the viability of cells following encapsulation with a Live/Dead viability reporter pair. Fluorescent staining following assay preparation confirmed cellular viability (Fig. 1b) while cell death was observed following boiling of the preparation (Fig. 1c).

**Fig. 2:**
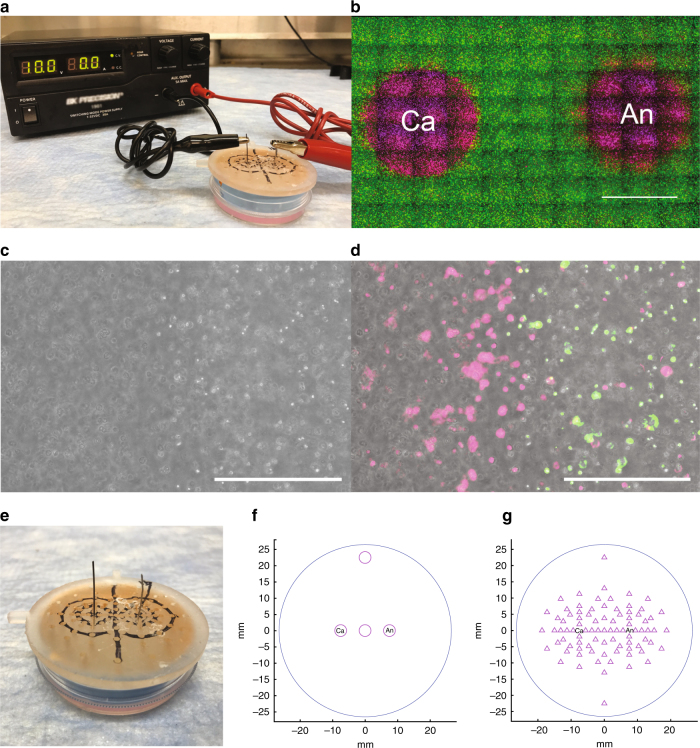
Use of a cell encapsulation assay enables the spatial resolution of electrolytic ablation-induced changes in temperature, transmembrane potential, and pH. **a** Electrolytic ablation was performed by the application of direct current between a nitinol cathode (left) and platinum anode (right) held in place by a 3D-printed spacer sitting atop the cell encapsulation matrix that was cast in a 6 cm tissue culture dish. **b** Electrolytic ablation performed in this assay led to the observation of cell death in the regions surrounding the cathode (Ca) and anode (An). Scale bar = 5 mm. **c**, **d** 10× magnification brightfield image with and without fluorescence reporter overlay at an increased cell density of 3 × 10^6^ cells mL^−1^ highlights changes at the border of the ablation zone. Scale bar = 400 μm. **e** A custom insert was 3D-printed to sit atop the encapsulation assay, which was prepared in a 6 cm dish. The insert holds the electrodes at a spacing of 1.5 cm and enables precise measurements of temperature, pH, and voltage potential (relative to the midpoint between the two electrodes). **f** Temperature measurement sites (circles). **g** pH and voltage potential measurement sites (triangles)

Electrolytic ablation was performed by applying a direct current between two inert needle electrodes placed 1.5 cm apart within encapsulation assays prepared in 6 cm tissue culture dishes (Fig. 2a, e). Initial investigation revealed that cell death occurred following ablation in the region surrounding both electrodes immediately following electrolytic ablation (Fig. 2b). The total area of cell death was dependent on the total time of ablation as well as the potential applied between the two electrodes. For the subsequent analyses, ablations were performed at a potential of 10 V with an electrode spacing of 1.5 cm. The duration of electrolytic ablation and the applied voltage were selected to ensure that appreciable cell death was observed between the cathode and anode; these settings corresponded to a total charge deposition of 726 mC for 90 s ablations (*n* = 3, SD = 41 mC) (Figs. 2b, 4k). At the viability margin, indicated by the fluorescent reporter pair, a corresponding change in the morphologic appearance of cells was appreciated on brightfield imaging where cells within the ablation margin appeared elongated, flattened, and pale in comparison to cells outside of this margin (Fig. 2c, d). These results were recapitulated in two additional HCC cell lines, SNU-449 and HEPG2, for which no significant difference in ablation area was observed (*F*: 2.29 on 2 and 11 DF, *p* = 0.15) (Supplementary Fig. 1).

### Characterization of the mechanism of cell death

**Fig. 3:**
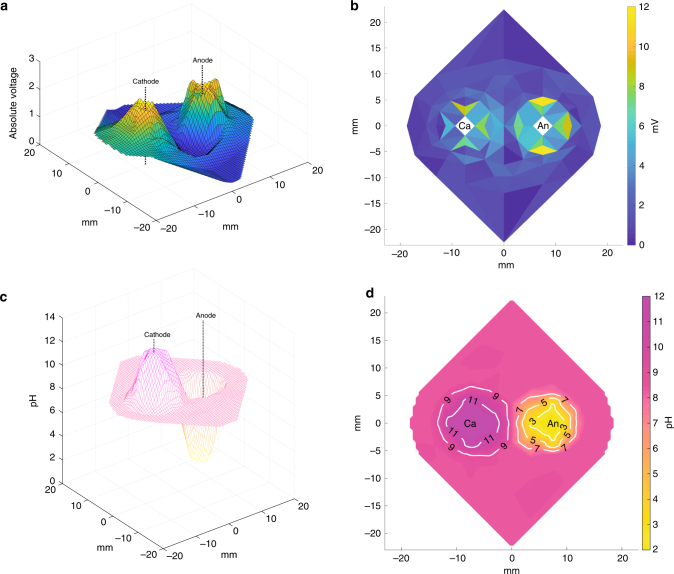
Measurement of voltage and pH in the encapsulation assay suggests the generation of acid and base as the mechanism of death following electrolytic ablation. **a** 3D surface plot of 2D linear interpolation of voltage potential measurements. **b** 2D linear interpolation contour plot of transmembrane potential calculated from voltage potential measurements via linear interpolation of the gradient magnitude across the width of an HCC cell. None of the regions of the assay reached the threshold voltage of 1 V necessary for electroporation. **c** 3D surface plot of 2D linear interpolation of pH measurements. **d** 2D linear interpolation contour plot of pH measurements recorded surrounding the cathode and anode, revealing basic changes surrounding the cathode [Ca] and acidic changes surrounding the anode [An]

Prior work led us to hypothesize that the cell death pattern observed after electrolytic ablation could be a function of temperature, transmembrane potential, or local changes in pH^34–36,38^. To further elucidate the mechanism of cell death following electrolytic ablation, we measured and analyzed these parameters independently within our model system either during or immediately following an ablation (Table 1, Fig. 3).

**Table 1:** Temperature measurements following electrolytic ablation reveal a temperature-independent mechanism of action

Anode	22 °C (*n* = 5, SD = 0)
Cathode	22 °C (*n* = 5, SD = 0.45)
Midpoint of electrodes	22 °C (*n* = 5, SD = 0.45)
Gel periphery	21 °C (*n* = 5, SD = 0)

Temperature measurements were made immediately following ablation at four sites surrounding the two electrodes (Fig. 2f)

Measurements of temperature at the anode, cathode, electrode midpoint, and a control measurement site outside of the zone of ablation were all found to fall between 21 and 23 °C (Fig. 2f, Table 1). Given the observation of cell death under these conditions without a corresponding change in temperature in the range expected of thermal ablation techniques, we concluded that temperature change was not the mechansim responsible for the observed pattern of death^3,4,6^. This finding is consistent with prior work and distinguishes this ablation technique from thermal-dependent modalities^36^.

Independent of temperature effects, transmembrane potential has been implicated as the cause of cell death in IRE with a transmembrane potential in excess of 1 V necessary to induce permanent membrane damage^10–13^. To calculate transmembrane potential, we first recorded the voltage potential at 85 sites surrounding the cathode and anode relative to the potential at the midpoint between the two electrodes (Figs. 2e, g, 3a). Next, the magnitude of a linearly discretized potential gradient was interpolated from these recorded measurements across a distance equal to the average cell diameter, 14.2 μm. The maximum recorded gradient was 12 mV and none of the regions of the cell encapsulation assay reached the threshold transmembrane potential of 1 V (Fig. 3b). Taken together, the absence of temperature change and electroporation-range transmembrane potentials distinguishes the mechanism of cell death in electrolytic ablation from those observed for thermal-based ablation modalities or IRE.

Given prior work demonstrating that electrolysis can yield reactive oxygen species (ROS), we subsequently investigated the potential role of ROS-mediated cell death in a modified version of our cell encapsulation platform^29,42^. Consistent with this prior work, we observed the formation of ROS surrounding both the anode and cathode. Treatment with 5 mM n-acetylcysteine (NAC), an antioxidant, led to an 88% decrease in this ROS burden. Viability assessments revealed a 23% decrease in the area of cell death after ablation with NAC treatment that did not reach statistical significance (*t*: 2.76 on 3.3 DF, *p* = 0.06) (Supplementary Fig. 2). This suggests that ROS may contribute to the cell death observed following electrolytic ablation but is not the primary driver of this phenomenon.

**Fig. 4:**
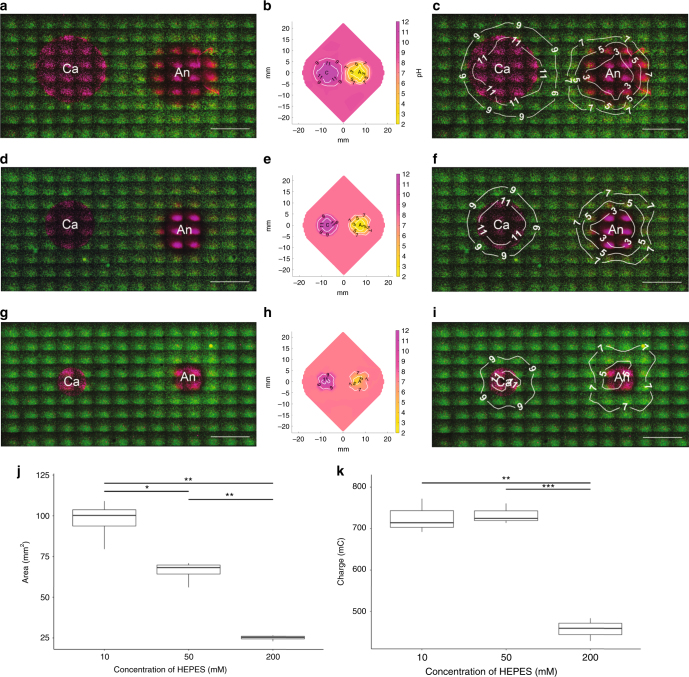
Modification of the buffering capacity confirms a pH-dependent mechanism of cell death in electrolytic ablation. **a**, **d**, **g** Viability images in the region surrounding the cathode and anode following electrolytic ablation with HEPES concentrations of 10, 50, and 200 mM. Scale bar = 5 mm. **b**, **e**, **h** pH contour maps in the region surrounding the cathode and anode following electrolytic ablation with HEPES concentrations of 10, 50, and 200 mM. **c**, **f**, **i** pH contour maps overlaid upon viability images in the region surrounding the cathode and anode following electrolytic ablation with HEPES concentrations of 10, 50, and 200 mM, respectively. Scale bar = 5 mm. **j** Comparison of the total area of cell death in the three conditions reveals a decreasing area of cell death with increased assay buffering capacity (*n* = 4 for all tests; [ANOVA] *F*: 75.96 on 2 and 9 DF, *p* < 1 × 10^−5^; [10 mM v 50 mM] *t*: 4.39 on 4.59 DF, Bonferroni adjusted *p* < .05; [10 mM v 200 mM] *t*: 11.36 on 3.10 DF, Bonferroni adjusted *p* < .01; [50 mM v 200 mM] *t*: 11.75 on 3.34 DF, Bonferroni adjusted *p* < .01). **k** Comparison of total charge deposition in the three conditions reveals a decreasing quantity of charge deposition with increased buffering capacity (*n* = 3 for all tests; [ANOVA] *F*: 72.38 on 2 and 6 DF, *p* < 1 × 10^−4^; [10 mM v 50 mM] *t*: −0.25 on 3.25 DF, Bonferroni adjusted *p*: 1.0; [10 mM v 200 mM] *t*: 9.36 on 3.48 DF, Bonferroni adjusted *p* < 0.01; [50 mM v 200 mM] *t*: 12.95 on 3.95 DF, Bonferroni adjusted *p* < 0.001)

In order to determine the primary driver of cell death following electrolytic ablation, we investigated the relationship of pH changes to the pattern of cell death in our assay. Measurements of pH were made immediately following ablation in 85 sites surrounding the anode and cathode (Fig. 2e, g). Reduction at the cathode leads to the generation of base, primarily via the generation of hydroxide ions (Eq. (1)). Oxidation at the anode leads to the generation of acidic species, primarily via the generation of hydronium ions (Eq. (2)). These pH changes were observed in our system and are consistent with prior ex vivo investigations of electrolytic ablation^34,37^ (Fig. 3c, d). Upon comparison to the viability data collected, we observed that pH contours paralleled the regions of cell death observed following viability staining of the cell encapsulation assay (Figs. 3c, d, 4).

1 $${\mathrm{Cathode}}\,{\mathrm{half}}\,{\mathrm{reaction}}\!:\quad 2{\mathrm{H}}_2{\mathrm{O}}{\mathrm{ + 2}}{{\mathrm{e}^ -}} \to {\mathrm{H}}_2{\mathrm{ + 2OH}}^ -$$

2 $${\mathrm{Anode}}\,{\mathrm{half}}\,{\mathrm{reaction}}\!:\quad 6{\mathrm{H}}_2{\mathrm{O}} \to {\mathrm{4H}}_3{{\mathrm{O}^ +}} {\mathrm{ + }}{\mathrm{O}}_2{\mathrm{ + 4}}{{\mathrm{e}^ -}}$$

### Validation of pH modulation as the driver of cell death

To further examine the role of pH change in electrolytic ablation-mediated cell death and macroscopic tumor necrosis, we modified the buffering capacity of the encapsulation assay prior to ablation. Initial experiments were performed with a 10 mM concentration of HEPES, a buffering agent used in cell culture to maintain a physiologic pH. We increased this buffering capacity of the system by factors of 5 and 20, preparing cell encapsulation assays with final concentrations of 50 and 200 mM HEPES, respectively, and repeated the measurements of pH and viability following electrolytic ablation (Fig. 4). The area of cell death was found to be significantly different in the three conditions (*F*: 75.96 on 2 and 9 DF, *p* < 1 × 10^−5^). Increased buffering capacity led to a contraction of the region of cell death (Fig. 4j). This change in viability correlated with a corresponding change in pH contours, whereby a reduced change in pH was associated with increased buffering capacity. Total charge delivery during electrolytic ablation also differed across the three experimental conditions, with increased buffering capacity leading to a reduced total charge delivery via an apparent increase in electrical impedance (*F*: 72.38 on 2 and 6 DF, *p* < 1 × 10^−4^) (Fig. 4k). Paired comparison of viability and pH data from the three conditions revealed the range of pH that was compatible with cellular viability. This range was observed to remain constant despite differences in the total ablated area following electrolytic ablation. pH above 10.6 (SD = 0.4) around the cathode or below 4.8 (SD = 0.6) around the anode was found to be incompatible with cellular viability (Table 2).

**Table 2:** Electrolytic ablation performed with differing buffering capacities reveals pH-dependent viability boundaries surrounding the two electrodes

	Cathode viability boundary (pH)	Anode viability boundary (pH)
10 mM HEPES	11.0	4.6
50 mM HEPES	10.2	4.2
200 mM HEPES	10.7	5.4
	10.6 (SD = 0.4)	4.8 (SD = 0.6)

The viability boundary for each experimental condition was calculated by determining the average pH along the boundary of an ellipse fitted to the viability images analyzed for each condition. pH below the level achieved at the anode viability boundary or above the level achieved at the cathode viability boundary was found to induce cell death in the encapsulation assay

**Fig. 5:**
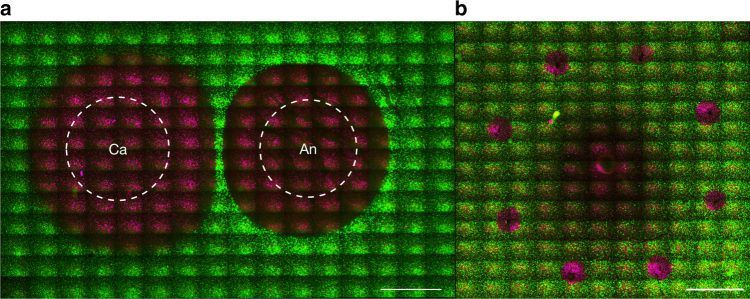
By permitting diffusion or using multiple cathodes, precise ablation geometries may be achieved with electrolytic ablation. **a** An increased area of cell death was observed after allowing 60 min of diffusion following electrolytic ablation (dashed white lines indicate ablation margin immediately after treatment; *t*: 7.32 on 3.4 DF, *p* < 0.01). Scale bar = 5 mm. **b** A multi-cathode design allows the prescription of the volume in which cell death occurs as demonstrated by performing electrolytic ablation at 10 V for 10 s resulting in cell death surrounding the nine electrodes. Scale bar = 5 mm

To examine the relative roles of pH and electrical charge in electrolytic ablation-induced cell death, we assessed the propagation of the ablation zone following the termination of the direct current application. While in the initial experiments, encapsulation assays were neutralized to a pH of 7.4 immediately prior to microscopy, we performed a subsequent experiment (*n* = 4) in which the assay was incubated at 37 °C for 60 min before neutralization and staining. Compared to the initial experimental condition, we observed an expansion in the total area of cell death consistent with diffusion of acidic and basic species in the absence of differences in electrical impedance or electric charge deposition (*t*: 7.32 on 3.42 DF, *p* < 0.01) (Fig. 5a). These findings indicate that electroosmosis is not responsible for cell death as the margins of the cellular necrosis propagated in the absence of an electric field. This propagation further supports the role of pH modulation as the primary driver of electrolytic ablation-induced cell death given that the expanded zone of cell death is consistent with the diffusion of acid and base generated at the electrodes.

### Leveraging the pH-dependent mechanism for HCC treatment

A primary advantage of electrolytic ablation is the enhanced precision it provides to shape ablation margins for the treatment of complex tumor geometries. The described experiments demonstrate that cell death induced through pH modulation can propagate within the encapsulation assay by diffusion of acidic and basic species. By accounting for tissue electric resistance and buffering capacity, electrolytic ablation could be designed to tailor the diffusion of pH fronts to the boundaries of interest. To further facilitate enhanced precision and demonstrate the capacity of electrolytic ablation to shape an ablation zone, we next performed a multi-cathode ablation with eight cathodes surrounding a central anode. We observed regions of cell death surrounding each of the nine electrodes in the geometry prescribed by the electrode arrangement, thus confirming the potential to shape the ablation zone (Fig. 5a, b).

## Discussion

These results demonstrate that electrolytic ablation-mediated cell death results primarily from the introduction of toxic acid and base. This occurs without evidence of significant temperature change or sufficient transmembrane potential to induce cell rupture, confirming a unique mechanism of action and distinguishing electrolytic ablation as the only ablation technology to induce its therapeutic effect through alteration of the tumor microenvironment^34,35,38,43,44^. Though not statistically significant, the reduction in the area of cell death following electrolytic ablation performed in the presence of an antioxidant suggests a potential secondary role of ROS in electrolytic ablation-induced cell death. However, the relatively low magnitude of this change affirms pH as the primary driver of cell death. Moreover, we demonstrate that a pH below 4.8 or above 10.6 is toxic to cancer cells, consistent with the range of pH changes observed in the region surrounding the cathode and anode in prior in vitro and in vivo studies^35,45^. The observation that increased buffering capacity led to the contraction of ablation zones in the setting of decreased charge deposition suggests that increased buffering capacity may slow ablation progress by increasing impedance. However, the observation that ablation proceeds following the cessation of electrolytic ablation in the absence of charge deposition underscores the primary role of pH modulation in causing cell death through the diffusion of acidic and basic species. The observed expansion of ablation zones in the absence of an electric field further confirms that cell death occurs independently of possible electroosmotic effects. Thus, the contraction of the ablation zone for assays with increased buffering capacity is due in part to the pH neutralizing influence of the buffer. In vivo, it is expected that physiologic buffering will limit the spread of acidic and basic species following the completion of treatment. This implies that knowledge of tissue impedance and local buffering capacity may be leveraged to sculpt the zone of ablation.

Thermal ablation is a curative therapy for solitary, primary tumors and is a treatment of choice in HCC for patients who are not surgical candidates^46,47^. RFA is the most widely implemented modality; however, like other hyperthermic techniques, it has limited success for tumors greater than 3 cm in diameter^48,49^. The primary drawback of hyperthermic ablation is the heat sink phenomenon whereby traversing vessels lead to non-spherical ablation zones and the need for increased ablative power yields off-target effects on the nearby healthy tissue^50,51^. This limitation contributes more broadly to the risk of ablation failure, local tumor progression, and post-treatment recurrence. Existing hypothermic and non-thermal ablation modalities, including cryoablation and IRE, are not limited by this phenomenon but are associated with potentially serious side effects. Cryoablation has been shown to precipitate a cryoshock phenomenon, a devastating condition that can lead to disseminated intravascular coagulation, acute respiratory distress syndrome, or liver failure^52^. IRE is technically difficult to perform and has the potential to induce cardiac arrhythmias and muscle contractions^3^.

Electrolytic ablation represents a promising non-thermal ablation strategy that is immune to the heat sink effects that limit thermal approaches. It uses relatively low electric potentials compared to IRE, which may improve the expected side effect profile^3^. Moreover, we have demonstrated that electrolytic ablation enables the unique capability to sculpt the zone of ablation based on the structure and arrangement of electrodes, permitting complex ablation margins with a single treatment. Clinically, this provides the potential to ablate lesions close to structures that may preclude heat-based ablation therapies—such as the gallbladder, porta hepatis, and bile ducts^38^. According to a model developed through simulations of pH front tracking, future work will be directed at developing a closed-loop system that evaluates ablation progress as a function of delivered charge and measured tissue impedance^35,37,40^. This may hold important implications for the implementation of gene electrotransfer, where knowledge of pH front patterns is necessary to optimize delivery^40^.

The primary limitation of the present work is that it was conducted in an in vitro tissue culture assay. As a result, in vivo ablations may not take place under the voltage, current, and temporal parameters described herein. In the present study, when buffering capacity was increased, deposited charge decreased for equivalent ablation lengths (Fig. 4). The extent of physiologic buffering and electrical resistance encountered in vivo may increase the duration of ablation required. Finally, the described assays do not assess the effect of tumor vasculature on ablation effectiveness. The flow of blood through a tissue may reduce the extent of ablation due to the delivery of additional buffering species and removal of generated acidic and basic ions. As such, the impact of blood flow in vivo is an important consideration for future translation.

In summary, we demonstrate that electrolytic ablation generates tumor cell killing through modulation of the tumor microenvironment primarily involving a pH-dependent mechanism that mitigates the limitations of leading ablation modalities. Our data further demonstrate a pH-threshold viability phenomenon wherein cancer cells placed in an environment at a pH below 4.8 or above 10.6 undergo cell death. These unique insights will be essential for leveraging cancer cell susceptibility to altered microenvironments as well as furthering the development of electrolytic ablation for clinical application.

## Methods

### Cell encapsulation

All studies were conducted with Huh-7, HepG2, or SNU-449 HCC cells grown to confluence with RPMI growth medium. Using 0.25% trypsin, cells were brought into a single cell suspension at a concentration of 5e5 cells mL^−1^. A homogenous 3% solution of low melting temperature agarose (Sigma A4018 2-Hydroxyethylagarose; Type VII) in water was prepared by heating. When the agarose cooled to a temperature of 45 °C, a 4 mL 1:1 mixture of cell suspension in RPMI and low melting temperature agarose was cast in 6 cm cell culture plates at room temperature. Initial mechanistic evaluation experiments were performed with a total HEPES concentration of 10 mM. Subsequent experiments included conditions with final HEPES concentrations of 50 or 200 mM.

### Electrolytic ablation

Ablations were performed within the cell encapsulation assay between a platinum anode (Surepure Chemetals #1981, 0.5 mm diameter) and nitinol cathode (Alfa Aesar 44950G6, 0.5 mm diameter) at an electrode spacing of 1.5 cm. For the experiment described in Fig. 5, eight cathodes were radially oriented around a central anode at a radius of 1 cm. Ablations were performed using a BK Precision^®^ 1901 D.C. power supply set to a constant potential of 10 V. All ablations were performed for 90 s, with the exceptions of those performed for evaluation of ROS (45 s) and evaluation of diffusion (10 s).

### Viability assessment

Viability was assessed and quantified with the ThermoFisher LIVE/DEAD^®^ Viability/Cytotoxicity Kit, for mammalian cells (L3224). Conversion of non-fluorescent, cell-permeable calcein AM to green-fluorescent calcein by intracellular esterases distinguishes living cells. Dead cells are distinguished by a red fluorescence when Ethd-1 enters cells with damaged membranes and binds to nucleic acid.

Following electrolytic ablation, 3 mL of 1M HEPES (pH = 7.4) was added on top of the cell encapsulation gels, which were then incubated for 30 min at 37 °C to allow for equilibration, which was verified with the pH probe described below. Upon removal of the buffer, 550 μL of staining solution composed of 2 μM calcein AM and 4 μM EthD-1 suspended in phosphate-buffered saline (PBS) was added to the gels, which were again incubated for 30 min at 37 °C. Fluorescence imaging was then performed on a Leica DMI6000B inverted light and fluorescent microscope. Using tiled acquisition, a series of fluorescence images were taken to capture the viability of the area surrounding both electrodes. Raw image files were prepared for quantification using Adobe Photoshop. Identical modifications were made to all acquired images. Quantification of the area of necrosis in the different experimental conditions was performed using ImageJ software. For the reported analyses, there were four replicates of each of the 10, 50, and 200 mM HEPES conditions. There were three replicates of the assessment of viability in the presence or absence of NAC. The brightfield and corresponding fluorescence images of Fig. 2c and d were acquired using a ThermoFisher EVOS FL Cell Imaging System. Green/red fluorescence was recolored as green/magenta using Adobe Photoshop for use in the presented figures.

### pH measurement

To determine the potential impact of pH on cellular viability in the described experiments, pH measurements were collected from 85 sites surrounding the two electrodes immediately following electrolytic ablation using a 3D printed part. pH was measured with the Orion^™^ 9863BN Micro pH Electrode (Thermo Fisher) on an A211 Benchtop meter (Thermo Fisher). Immediately following electrolytic ablation, the pH probe was used to measure the pH of up to 10 sites. Given the concern for rapid pH changes secondary to diffusion, measurements of pH within a single assay were performed within the first minute following the conclusion of ablation. This process was repeated on multiple assays to sample all 85 points at least twice for each of the three experimental conditions.

### Local voltage and transmembrane potential

Analogous to pH measurements, voltage potentials within the cell encapsulation assay were collected from 85 sites surrounding the two electrodes. Voltage recordings were measured on the Peakmeter PM18C multimeter relative to the platinum anode with a second nitinol needle electrode. At the time of voltage measurement, the two electrodes of the assay were connected to the 10 V power supply. Up to 10 measurements were collected from each assay, and all 85 sites were sampled at least four times. Raw measurements of potential were then re-centered around the midpoint connecting the two electrodes. The magnitude of this re-centered measurement is reported as the potential of a site within the assay relative to the midpoint between the two electrodes. Transmembrane potential was estimated by 2D linear interpolation of voltage between measured points followed by numerical calculation of gradient magnitude over a distance corresponding to the average diameter of a cell used in the encapsulation assay, 14.2 μm. This size was determined using a Countess II FL Automated Cell Counter.

### Current measurement

Current measurements were acquired with the TACKlife DM01M digital multimeter. Measurements of current were recorded every 10 s during electrolytic ablation and the total charge delivered was subsequently calculated. These measurements were repeated three times for each experimental condition.

### Temperature measurement

The temperature was recorded using the Peakmeter PM18C multimeter. Measurements of temperature were taken immediately following electrolytic ablation at the anode, cathode, and the midpoint between the two electrodes. These measurements were repeated five times for the baseline experimental condition (10 mM HEPES, Huh7 cells).

### ROS assessment

Huh7 cells were grown and encapsulated as described above with the exception that 2 mLs, as opposed to 4 mLs, of the 1:1 mixture of encapsulated cells were plated onto 6 cm plates. Control plates were prepared with 1 mM H_2_O_2_ in PBS (positive control), 1 mM H_2_O_2_ with 5 mM NAC in PBS (negative control), or bland PBS (negative control) and were then incubated at 37 °C for 30 min. Subsequently, CellRox^®^ Green (Invitrogen, Carlsbad, CA) was added to the PBS fraction at a final concentration of 17.5 µM. The plate was gently agitated and incubated at 37 °C for 30 min. The liquid was aspirated and replaced with PBS, followed by a 15 min incubation at 37 °C. Finally, the liquid was aspirated and replaced with PBS.

To evaluate ROS generation, electrolytic ablation was performed at a potential of 10 V for 45 s. Prior to electrolytic ablation, assays were pre-incubated with 1 mL of PBS with or without 5 mM NAC and these solutions were aspirated prior to ablation. Immediately after electrolytic ablation, assays were incubated in 3 mL of 1M HEPES (pH = 7.4). After equilibration, this solution was replaced with 1 mL of PBS supplemented with CellRox^®^ Green at a final concentration of 17.5 µM with 5 mM NAC for those which had previously received NAC. Plates were incubated at 37 °C for 30 min. Assays were then washed once with PBS at 37 °C for 30 min. There were three replicates of the assessment of ROS in the presence or absence of NAC.

Fluorescence imaging was then performed on a Leica DMI6000B inverted light and fluorescent microscope. Using tiled acquisition, a series of fluorescence images were taken to capture the ROS in the area surrounding both electrodes. Raw image files were prepared for quantification using Adobe Photoshop. Identical modifications were made for all acquired images. For the reported analyses, fluorescence from the areas corresponding to both electrodes was compared with respect to the background of the image excluding the areas of the electrodes as a log fold change. This quantification was performed using ImageJ software.

### Statistical analyses and figure generation

Statistics were performed in R. *t*-tests between conditions were performed with two-tails assuming unequal variance between the different conditions with a Bonferroni correction for multiple *t*-tests. The results of *F*-tests and *t*-tests are reported above with corresponding statistics, degrees of freedom, and *p*-values. Quantitative displays were prepared in R and Matlab, with use of the export_fig plug-in (https://www.mathworks.com/matlabcentral/fileexchange/23629-export-fig). Figures were compiled using Adobe Illustrator. Adobe Photoshop was used to insert pH map overlays produced in Matlab.

### Code availability

The code used is available in the Dryad Digital Repository at 10.5061/dryad.2gg2f50^53^.

### Data availability

The datasets generated during and/or analyzed during the current study are available in the Dryad Digital Repository at 10.5061/dryad.2gg2f50^53^.

## Electronic supplementary material

Supplementary Information
