# Supplementary material for: Electrolytic ablation enables cancer cell targeting through pH modulation
Source: Commun Biol. 2018 May 17;1:48. doi: 10.1038/s42003-018-0047-1 (PMC6123816; doi:10.1038/s42003-018-0047-1)
Supplement: Supplementary file 1 — Supplementary Information [file 42003_2018_47_MOESM1_ESM.pdf]

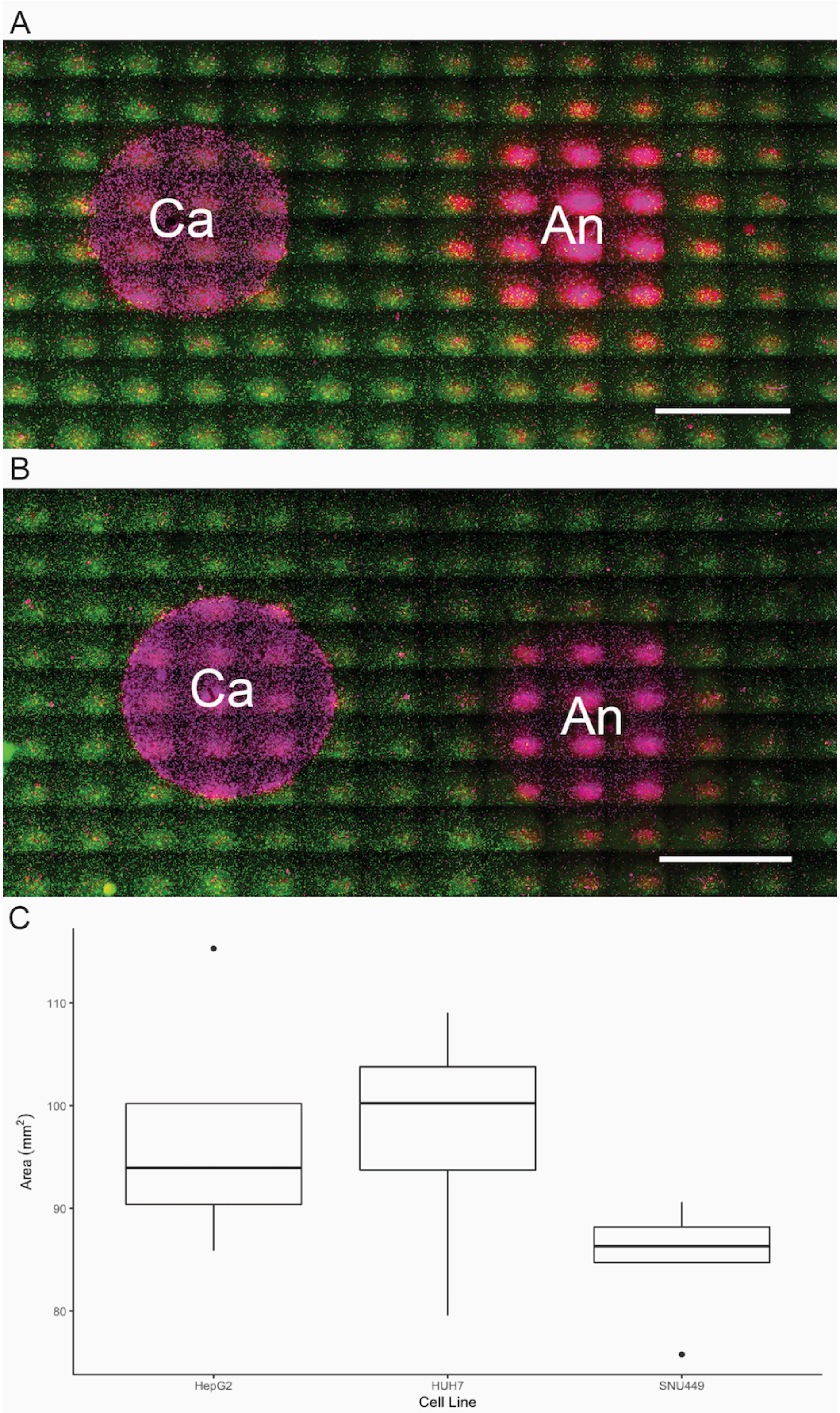

Supplemental Figure 1 | Electrolytic ablation is found to induce cell death in multiple HCC cell lines
a, Electrolytic ablation demonstrates evidence of cell death surrounding the cathode (Ca) and anode (An) in SNU-449 cells [Scale bar = 5 mm]. **b,** Electrolytic ablation demonstrates evidence of cell death surrounding the cathode (Ca) and anode (An) in HepG2 cells [Scale bar = 5 mm]. **c,** Comparison of the area of cell death observed in three HCC cell lines reveals no significant difference in response to electrolytic ablation (F: 2.29 on 2 and 11 DF, $p = 0.15$).

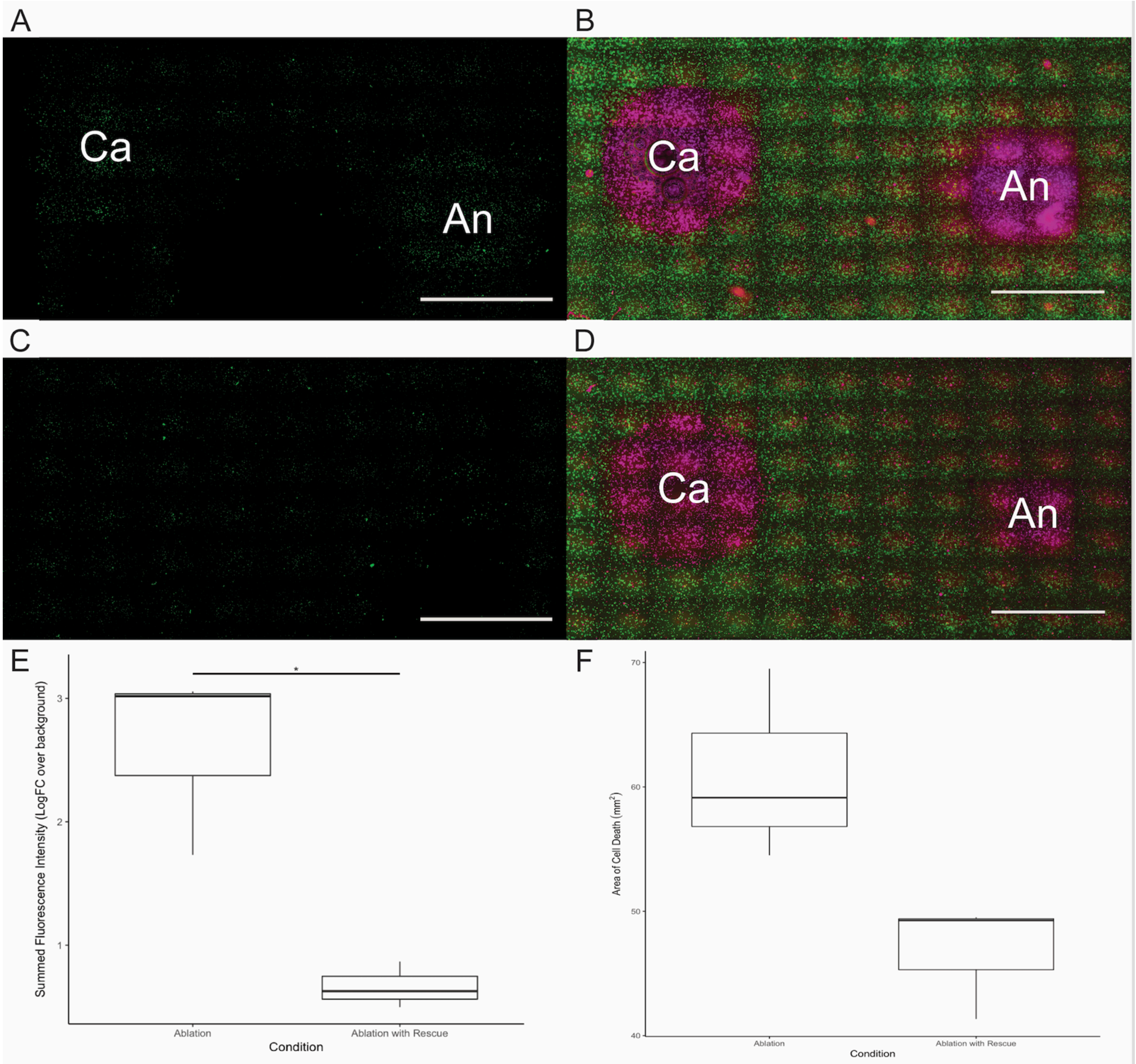

Supplemental Figure 2 | Reactive oxygen species are present following electrolytic ablation
a, Following electrolytic ablation, assays stained with CellRox® Green demonstrated areas of fluorescence in the regions surrounding the cathode (Ca) and anode (An), suggesting the presence of ROS. [Scale bar = 5 mm] **c,** Exposure to 5 mM NAC, an antioxidant, reduced the amount of fluorescence detected from the regions surrounding the electrodes. [Scale bar = 5 mm] **b, d,** Viability staining with Calcein/Ethd-1 reveals cell death in the absence (c) or presence (d) of NAC. [Scale bar = 5 mm] **e,** Summed fluorescence intensity around the two electrodes, compared according to the natural log fold change with respect to the background of the image, demonstrated a significant decrease in ROS detection (t: 4.32 on 2.2 DF, $p < 0.05$). **f,** The mean area of cell death observed after electrolytic ablation following rescue with NAC decreased by 23%, though this change did not reach statistical significance (t: 2.76 on 3.3 DF, $p = 0.06$).
